# Characterization of *Sonic Hedgehog* transcripts in the adult mouse brain: co-expression with neuronal and oligodendroglial markers

**DOI:** 10.1007/s00429-023-02756-2

**Published:** 2024-02-08

**Authors:** Mariagiovanna Russo, Giuliana Pellegrino, Hélène Faure, Linda Tirou, Ariane Sharif, Martial Ruat

**Affiliations:** 1 CNRS, Paris-Saclay University, UMR-9197, Neuroscience Paris-Saclay Institute, 91400 Saclay, France; 2 Univ. Lille, Inserm, CHU Lille, Laboratory of Development and Plasticity of the Neuroendocrine Brain, Lille Neuroscience and Cognition, UMR-S 1172, FHU 1000 Days for Health, Lille, France

**Keywords:** RNAscope, Neurodegenerative diseases, Multiple sclerosis, Patched, Shh, Smoothened

## Abstract

In the adult mammalian brain, astrocytes are proposed to be the major Sonic Hedgehog (Shh)-responsive cells. However, the sources of the Shh molecule mediating activation of the pathway are still poorly characterized. The present work investigates the distribution and phenotype of cells expressing Shh mRNA in the adult mouse brain. Using single-molecule fluorescent in situ hybridization (smfISH), we report much broader expression of Shh transcripts in almost all brain regions than originally reported. We identify Shh mRNA in HuC/D^+^ neuronal populations, including GABAergic (glutamic acid decarboxylase 67, Gad67), cholinergic (choline acetyltransferase, ChAT), dopaminergic (tyrosine hydroxylase, TH), nitrergic (neuronal nitric oxide synthase, nNOS), and in a small population of oligodendroglial cells expressing Sox10 and Olig2 mRNA transcription factors. Further analysis of Shh mRNA in cerebral cortical and hypothalamic neurons suggests that Shh is also expressed by glutamatergic neurons. Interestingly, we did not observe substantial Desert Hedgehog and Indian Hedgehog mRNA signals, nor Shh signals in S100β^+^ astrocytes and Iba1^+^ microglial cells. Collectively, the present work provides the most robust central map of Shh-expressing cells to date and underscores the importance of nitrergic neurons in regulating Shh availability to brain cells. Thus, our study provides a framework for future experiments aimed at better understanding of the functions of Shh signaling in the brain in normal and pathological states, and the characterization of novel regulatory mechanisms of the signaling pathway.

**Supplementary Information:**

The online version contains supplementary material available at 10.1007/s00429-023-02756-2.

## Introduction

The early discovery of Sonic Hedgehog (Shh) transcript expression in mature rodent brain neurons (Traiffort et al. 1998, 1999) paved the way for further studies on the role of Shh signaling in the mammalian brain under physiological conditions or during disease (Briscoe and Therond 2013; Ferent et al. 2013b; Alvarez-Buylla and Ihrie 2014; Ruat et al. 2015; Yao et al. 2016; Garcia et al. 2018; Laouarem and Traiffort 2018; Garcia 2021; Laouarem et al. 2021). Studies in rodents have highlighted a primary role for the pathway in regulating neurogenic niches in the subventricular zone of the lateral ventricles (SVZ) and the subgranular zone (SGZ) of the hippocampus, and the status of oligodendroglial progenitors in brain and cortical structures. Positive modulation of the Shh signaling pathway has been proposed as a potential therapeutic approach for Parkinson's disease (Gonzalez-Reyes et al. 2012; Malave et al. 2021) and for demyelinating diseases (Loulier et al. 2006; Ferent et al. 2013b; Porcu et al. 2015; Samanta et al. 2015; Del Giovane et al. 2022). Blocking the pathway by small molecules inhibitors of the GTP-binding protein-coupled receptor Smoothened (Smo) is proposed as an alternative for treating Shh-dependent medulloblastoma, a cerebellar tumor with a poor prognosis in children (Ruat et al. 2014; Swiderska-Syn et al. 2022). Genetic mutations affecting genes associated with Shh signaling are responsible for the developmental diseases such as holoprosencephaly or medulloblastoma (Wolter et al. 1997; Reifenberger et al. 1998; Traiffort et al. 2004; Ruat et al. 2015; Andreu-Cervera et al. 2021).

Shh belongs to a family of secreted morphogens including Desert Hedgehog (Dhh) and Indian Hedgehog (Ihh). These molecules are synthesized as precursors that undergo internal cleavage generating an aminoterminal peptide displaying almost all biological activities of the peptides. Binding of Shh to its receptor Patched (Ptc), a member of the Resistance-Nodulation-Division transporter superfamily, activates Smo (Ruat et al. 2014; Zhang and Beachy 2023). These events result in complex modulation of the zinc-finger transcription factors glioma-associated oncogenes 1–3 (Gli1–3). Shh is widely distributed in rodent brain regions, whereas Dhh and Ihh expression has not been detected (Ruat et al. 2015; Garcia et al. 2018). Astrocytes have been proposed as the primary targets of Shh signaling as they express components of the Shh transduction machinery including Ptc, Smo, and Gli1–3 (Garcia et al. 2010, 2018; Ruat et al. 2015; Allahyari et al. 2019; Hill et al. 2019; Tirou et al. 2021; Wang et al. 2021). However, the discrepancy between the expression of Ptc and Smo transcripts in several brain areas has led to the hypothesis that Ptc may transduce the Shh signal in the absence of Smo and that Smo may be activated by non-canonical mechanisms (Traiffort et al. 1998; Charytoniuk et al. 2002). It has been reported by us and other groups that both Ptc and Smo are expressed in neurons (Alvarez-Buylla and Ihrie 2014; Ruat et al. 2015; Garcia et al. 2018). The initial discovery of cells expressing Shh transcripts using specific digoxigenin-labeled riboprobes in rodent brain (Traiffort et al. 1998) was followed by detailed analysis of cells expressing this marker in mature rodent central nervous system under physiological and pathophysiological conditions. These studies were conducted with Shh-reporter mouse lines, Shh antibodies, or radioactive and cold riboprobes (Traiffort et al. 1998; Traiffort et al. 2001; Charytoniuk et al. 2002; Lai et al. 2003; Machold et al. 2003; Desouza et al. 2011; Ihrie et al. 2011; Eitan et al. 2016; Sanchez and Armstrong 2018; Sanchez et al. 2018; Gonzalez-Reyes et al. 2019; Rivell et al. 2019; Tirou et al. 2020; Tu et al. 2023). Thus, it has been proposed that Shh is synthesized by a subpopulation of GABAergic cells expressing Gad67 in the ventral brain, by a restricted population of cholinergic neurons expressing ChAT in the motor nuclei and in the basal forebrain (Traiffort et al. 1999; Pascual et al. 2005; Ihrie et al. 2011), by cerebral cortical neurons mainly located in layer V of the cerebral cortex (Charytoniuk et al. 2002; Harwell et al. 2012), in dopaminergic neurons of the substantia nigra (Gonzalez-Reyes et al. 2012; Turcato et al. 2022), in a population of hilar cells (McMahon et al. 2003; Gonzalez-Reyes et al. 2019), in some Pomc^+^ cells of the arcuate nucleus (ARC) and some Mchr1^+^ cells of the paraventricular nucleus (PVN) in the hypothalamus (Antonellis et al. 2021), and in Purkinje cells of the cerebellum (Traiffort et al. 1998; Wallace 1999; Farmer et al. 2016). Several ventral brain regions, including the hypothalamic and thalamic nuclei, have also been reported to express Shh signaling (Garcia et al. 2010; Antonellis et al. 2021; Tirou et al. 2021). In most of these studies, Shh is expressed by neurons, while Shh expression in astrocytes and microglia has been observed occasionally in physiological conditions (Sanchez and Armstrong 2018); whereas, Shh expression in these cells under pathophysiological conditions required further investigations (Ruat et al. 2015; Yao et al. 2016; Garcia et al. 2018; Laouarem and Traiffort 2018; Garcia 2021).

Recently, we reported broad expression of Shh recognized by the specific monoclonal antibody C9C5 in a subset of mature CC1^+^ oligodendrocytes (Tirou et al. 2020). We also identified Shh transcripts by single-molecule fluorescent in situ hybridization (smfISH) in a subset of cells expressing the oligodendroglial markers Olig2 and Sox10 mRNA. These results revealed that Shh expression was more extensive than originally reported. Now, we report a much broader expression of Shh transcripts in the mature mouse brain, which occurs in diverse neuronal populations and in a restricted population of oligodendroglial cells. We also identified a new population of neurons co-expressing Shh transcripts and the nitrergic marker nNOS and reported broad expression of Shh transcripts in hypothalamic nuclei, suggesting potential new roles for Shh in the regulation of neural circuits.

## Materials and methods

### Ethical approval

All animal experiments were performed in accordance with the Council Directive 2010/63EU of the European Parliament and were approved (Project No. 4558) by the French ethic committee.

### Mice and tissue preparation

Young adult (2–3 months old) rodent have been used previously for reporting expression of Shh or its signaling components (Ruat et al. 2015; Laouarem et al. 2018; Garcia 2021). Thus, young adult (2–3 months old) C57Bl/6J (Janvier-Labs) were housed grouped and maintained with access to food and water ad libitum. The room had constant temperature (21–22 °C) and a 12:12-h light/dark cycle. Mice were deeply anesthetized with intraperitoneal injection of xylazine 4 mg/kg (Sedaxylan, Chetra) and ketamine 20 mg/kg (Ketalar, Parke-Davis), and were perfused with 4% paraformaldehyde (PFA) in 0.2 M sodium phosphate, pH 7.4. After dissection, brains were post-fixed for 2 h in 4% PFA and cryoprotected in 20% sucrose/PBS waiting for the tissue to sink at the bottom of the tube. The tissues were mounted in Optimal Cutting Temperature (O.C.T.) compound Tissue-Tek (Sakura Finetek), frozen in liquid nitrogen-cooled isopentane and kept at − 80 °C until use. Tissues were allowed to equilibrate to − 20 °C in the cryostat (Leica, CM3050) for ~ 30 min and 14 μm coronal sections were mounted onto SuperFrost^®^ ultra Plus slides (Thermo Scientific), dried for 1–2 h at − 20 °C and stored at − 80 °C for less than three months. Anatomical landmarks were determined using a mouse brain atlas (Paxinos and Franklin 2001).

### RNAscope^®^ in situ multiplex fluorescent assay

The RNAscope Multiplex Fluorescent v2 Reagent Kit was used according to the manufacturer’s instructions (Advanced Cell Diagnostics) with minor modifications. Slides were post-fixed in 4% PFA for 30 min, and then washed in sterile PBS (2 × 5 min) followed by dehydration in increasing concentrations of ethanol (50%, 70%, 100%) for 5 min each at room temperature. Then, slides were air-dried for 5 min and incubated for 30 min at 37 °C in a humidified chamber. Sections were then pretreated with hydrogen peroxide (10 min) and antigen retrieval was performed for 15 min at 98 °C. After 5-min drying at room temperature, a hydrophobic barrier was drawn around the brain sections with ImmEdge Pen and they were dried again for 5 min. Sections were treated with RNAscope^®^ Protease Plus, and incubated with hybridization probes and amplifiers. Positive and negative control probes were run in each experiment as an internal control, to assess sample RNA quality and tissue optimal permeabilization. Development of HRP signal with TSA^®^ plus fluorophores was performed exactly as recommended with the dilution indicated in Table S1. Finally, slides were counterstained with RNAscope^®^ Multiplex Fl v2 DAPI. Images were acquired with a 20 × objective using an Axio Imager Z2 ApoTome microscope equipped with a motorized stage (Zeiss, Germany) or a fluorescence microscope Leica DM2000. All images were captured over a defined z-focus range corresponding to visible fluorescence within the section. Negative controls were used to assess the negative control background and to set the signal-to-noise ratio for background levels for each experiments. Positive controls were used to assess the positive control signal strength. Images were analyzed using ZEN software (Zeiss) or LAS-X software (Leica) and reconstructed in ImageJ 1.52p (NIH) using the z-stack module and Photoshop CS3 (Adobe).

### Immunohistochemistry

Some sections were subjected to immunohistochemistry after RNAscope and before counterstaining. They were blocked 1 h in PBS, 0.25% Triton-X100, 1% BSA. After washing in PBS pH 7.4 (2 × 5 min), the primary antibodies, detailed in Supplementary Information Table S2, were incubated at 4 °C overnight for anti-S100β and anti-HuC/D or for 2 nights for anti-TH. Next, slices were washed in PBS (3 × 5 min) and incubated with the secondary antibody (1/400, 488-conjugated Donkey, anti-mouse (# R37114) or anti rabbit (#R37118), Invitrogen) for 2 h at room temperature. Finally, slides were washed again in PBS (3 × 5 min) and counterstained with RNAscope^®^ Multiplex Fl v2 DAPI to be analyzed as described above. Association of in situ probes and antibodies is listed in Supplementary Information Table S3 together with sagittal position to bregma of the sections analyzed.

### Cell count and analysis

Brain regions were delineated using the Allen Adult Mouse Brain Atlas (http://atlas.brain-map.org/atlas?atlas=602630314#atlas=602630314&). The nomenclature used corresponds to the one described in the Paxinos Atlas. The quantification of HuC/D-, *Gad67*-, *nNOS*-, and TH-expressing neurons was done by counting the number of single labeled cells in the arcuate (ARC), ventromedial (VMH) and dorsomedial (DMH) hypothalamic nuclei, represented by plate 70 of the Allen Adult Mouse Brain Atlas, and in the substantia nigra compacta (SNc) and ventral tegmental area (VTA), represented by plate 82 of the same Atlas. Cells were counted unilaterally in the ARC, VMH, DMH, SNc and VTA delimited by DAPI nuclear staining from the analysis of one section per adult mouse, *N* = 3 animals. Between 100 and 170 cells were counted as TH-expressing neurons in the SNc and VTA nuclei. From 100 to 400 HuC/D-expressing cells were counted in the ARC, VMH, and DMH. Between 100 and 200 cells were counted as *Gad67*-expressing neurons in the ARC and DMH; while from 9 to 18 *Gad67*-expressing neurons were counted in the VMH, where *Gad67* transcript is less expressed. From 100 to 225 *nNOS*-expressing cells were counted in the VMH and DMH; while 42–48 cells were counted in the ARC where the *nNOS* transcript is less expressed. Density of Shh mRNA was estimated by RNAscope from one section per adult mouse, * N* = 3 animals, and scored as follows: 4^+^: very high density, > 30 dots/cell; 3^+^: high density, 10–30 dots/cell; 2^+^: moderate density, 6–9 dots/cell; 1^+^: low density, 2–5 dots/cell; 0^+^: undetectable, 0–1 dot/cell. Counting of colocalized staining was undertaken using ROI and multi-point ImageJ tools. Data are represented as the mean ± SEM.

## Results

### Shh mRNA expression is widespread in the brain, whereas Dhh and Ihh mRNAs are not

**Fig. 1:**
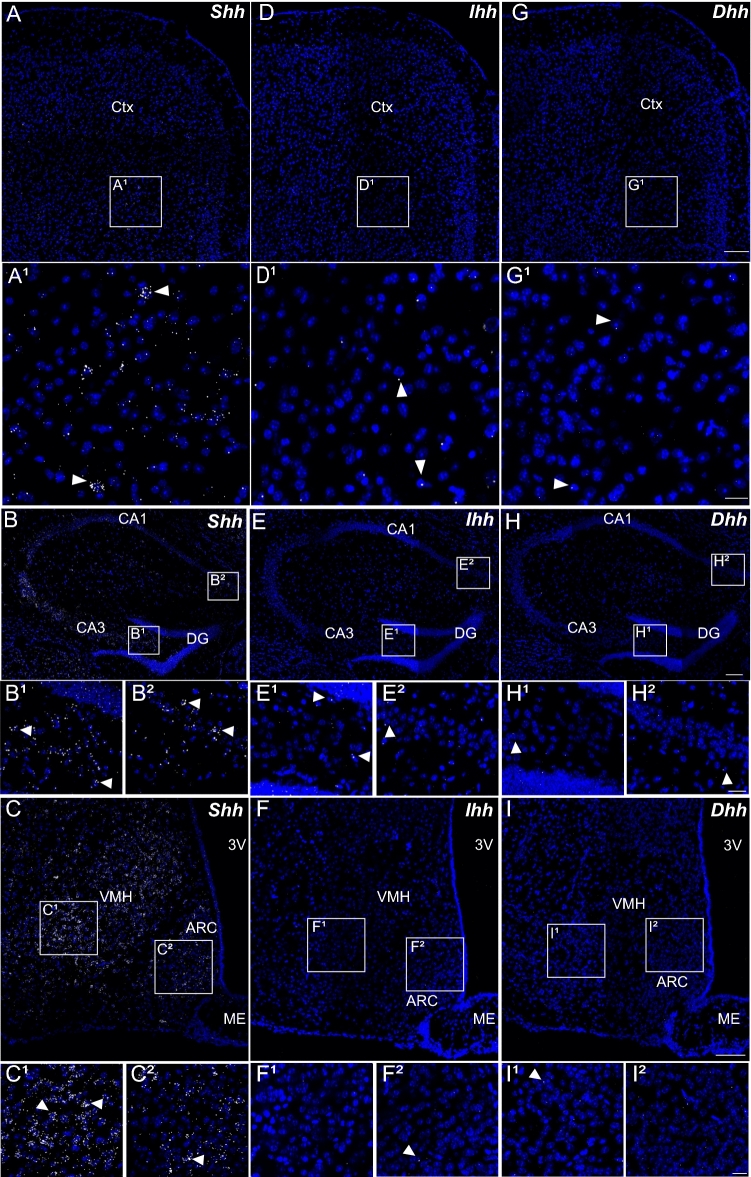
Representative images of Shh, Dhh, and Ihh transcripts distribution by in situ hybridization in distinct adult mouse brain regions. Shh transcripts are identified in the cerebral cortex (Ctx) (**A**), in various layers of the hippocampus (**B**) and in hypothalamic nuclei (**C**). The highest expression is observed in cells located in layer V of the cerebral cortex (**A1**), in CA3 (**B1**) and CA1 (**B2**) layers of the hippocampus, in the ventromedial hypothalamic nucleus (VMH) (**C1**) and the arcuate hypothalamic nucleus (ARC) (**C2**). Rare Ihh and Dhh transcripts are present in the cerebral cortex (**D**, **G**), in the hippocampus region (**E**, **H**), and in hypothalamic nuclei (**F**, **I**). **A1**–**I2** are high magnification views of indicated boxed areas in the main panels. Arrowheads show cells associated to one (Ihh, Dhh) or more (Shh) dots. Sections are counterstained using DAPI to visualize cell nuclei. *LV* lateral ventricle, *DG* dentate gyrus of the hippocampus, *ME* median eminence, *3V* third ventricle. Scale bars: **A**–**I** = 100 µm; **A1**–**I2** = 20 µm

We used single-molecule multiplex fluorescent in situ hybridization (smfISH) to study the distribution of Shh, Dhh, and Ihh mRNAs in the adult mouse brain (Fig. 1). We successfully detected consistent and reproducible expression of Shh transcripts using RNAscope in various brain regions (Fig. 1A–C). As shown in Table 1, the cell-associated density of Shh mRNA was assessed from the number of spots counted per nucleus and classified as "very high density," "high density," "moderate density," "low density," and "undetectable." Regions expressing very high density of Shh signals were found in the striatum, pallidum, claustrum, trigeminal mesencephalic and motor nuclei, facial nucleus, and in the Purkinje cell layer of the cerebellum, as previously reported using a digoxigenin-labeled Shh-specific cRNA probe (Traiffort et al. 1998, 1999, 2001). However, most brain regions had cells expressing Shh mRNA at low to moderate or low to high density, whereas fibrous tracts and a few nuclei expressed cells at low density only (Table 1). Probes for Dhh and Ihh, the other two members of the Hedgehog (Hh) family (Briscoe and Therond 2013) did not generate significant signals in adjacent brain sections by RNAscope: one dot per nucleus was occasionally observed with both probes as shown in the cerebral cortex, hippocampus, and in the tuberal region of the hypothalamus (Fig. 1D–I).

**Table 1:** Distribution of *Shh* transcripts in the mouse brain

Region	Nucleus		
Cortex	Layer I		1^+^
	Layer II/III		1^+^–3^+^
	Layer IV		1^+^–2^+^
	Layer V		1^+^–3^+^
	Layer VIa		1^+^–3^+^
	Layer VIb		1^+^–3^+^
	Claustrum		1^+^–4^+^
Hippocampus	CA1		1^+^–3^+^
	CA2		1^+^–3^+^
	CA3		1^+^–3^+^
	Dentate gyrus		
		Granul cell layer	1^+^–3^+^
		Polymorph layer	1^+^–2^+^
		Molecular layer	1^+^–2^+^
Striatum	Caudate putamen		1^+^–4^+^
	Anterior amygdala area		1^+^–4^+^
Pallidum	Globus pallidus		1^+^–4^+^
	Magnocellular preoptic nucleus		1^+^–4^+^
	Medial septal nucleus		1^+^–3^+^
	Lateral septal nucleus		1^+^–4^+^
Thalamus	Medial habenular nucleus		1^+^
	Lateral habenular nucleus		1^+^–3^+^
	Paraventricular nucleus		1^+^–3^+^
	Mediodorsal nucleus		1^+^–3^+^
	Intermediodorsal nucleus		1^+^–3^+^
	Centrolateral nucleus		1^+^–3^+^
	Paracentral nucleus		1^+^–2^+^
	Central medial nucleus		1^+^–3^+^
	Lateral posterior nucleus		1^+^–3^+^
	Dorsolateral geniculate nucleus		1^+^
	Lateral dorsal nucleus		1^+^
	Posterior nucleus		1^+^–3^+^
	Ventral nucleus		
		Antero-lateral	1^+^–2^+^
		Postero-lateral	1^+^
		Medial	1^+^–3^+^
		Postero-medial	1^+^–2^+^
	Reticular nucleus		1^+^
	Rhomboid nucleus		1^+^–3^+^
	Submedius nucleus		1^+^–3^+^
	Xiphoid nucleus		1^+^–2^+^
	Reuniens nucleus		1^+^–2^+^
	Ventral reuniens nucleus		1^+^–2^+^
Hypothalamus	Arcuate hypothalamic nucleus		
		Dorsal part	1^+^–3^+^
		Ventral part	1^+^–3^+^
	Ventromedial hypothalamic nucleus		
		Dorsomedial part	1^+^–3^+^
		Central part	1^+^–3^+^
		Ventromedial part	1^+^–3^+^
	Dorsomedial hypothalamic nucleus		1^+^–3^+^
	Posterior hypothalamic nucleus		1^+^–3^+^
	Lateral hypothalamic nucleus		
		Anterior	1^+^–3^+^
		Posterior	1^+^–3^+^
	Median eminence		1^+^
	Tuberal nucleus		1^+^–2^+^
	Anterior hypothalamic nucleus		1^+^–2^+^
	Sub-paraventricular zone		1^+^–3^+^
	Paraventricular hypothalamic nucleus		1^+^–3^+^
	Suprachiasmatic nucleus		1^+^
	Optic chiasma		1^+^
	Mammillary body		1^+^–3^+^
Midbrain	Ventral tegmental area		1^+^–3^+^
	Substantia nigra, pars compacta		1^+^–3^+^
	Substantia nigra, pars reticulata		1^+^–3^+^
Pons	Mesencephalic trigeminal nucleus		1^+^–4^+^
	Pontine central gray		1^+^–3^+^
	Motor trigeminal nucleus		1^+^–4^+^
Medulla	Facial nucleus		1^+^–4^+^
	Intermediate reticular nucleus		1^+^–3^+^
	Gigantocellular reticular nucleus		1^+^–3^+^
	Parapyramidal nucleus		1^+^–2^+^
Cerebellum	Granule cell layer		1^+^
	Purkinje cell layer		4^+^
	Molecular layer		1^+^
	White matter		1^+^

Density of *Shh* mRNA was estimated by smfISH from *N* = 3–4 animals. 4 + : very high density, > 30 dots/cell; 3 + : high density, 10–30 dots/cell; 2 + : moderate density, 6–9 dots/cell; 1 + : low density, 2–5 dots/cell. Nuclei associated to one dot were observed scattered in all regions. *Shh*-transcript expression was considered as “undetectable” in these cells

### Shh mRNA is present in cells expressing neuronal and oligodendroglial markers

**Fig. 2:**
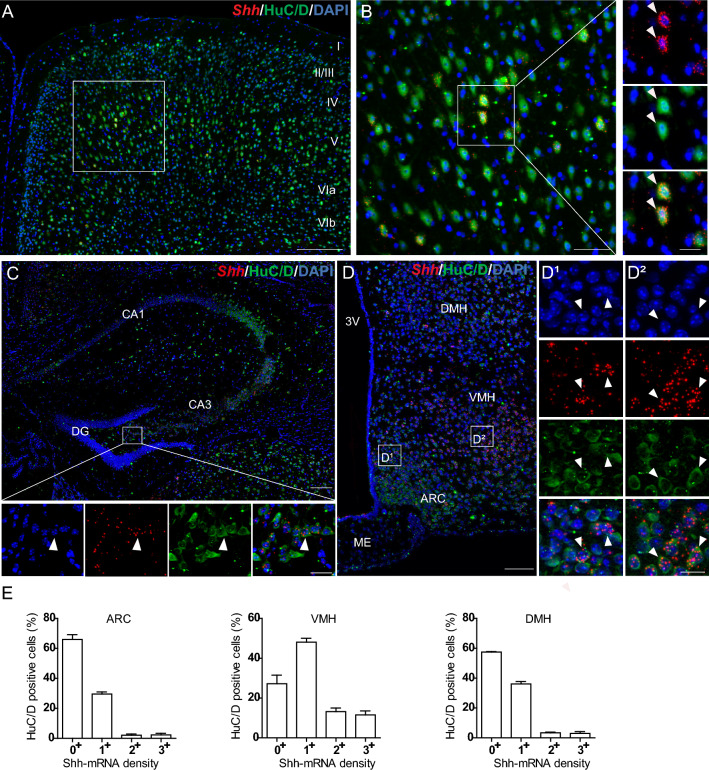
Representative images of fluorescent in situ hybridization of *Shh* (red) combined with immunofluorescence for the neuronal marker HuC/D (green), in mouse cerebral cortical layers (**A**, **B**), hippocampus (**C**) and mediobasal hypothalamus (**D**–**D2**). *Shh* is detected with varying intensity level in neurons expressing HuC/D showing the highest expression in cortical layer V (**B**), in the arcuate hypothalamic nucleus (ARC) and ventromedial hypothalamic nucleus (VMH) (**D1**–**D2**). **B** is the magnification of boxed area in **A**. White square in **C** is magnified below the main panel. Arrowheads show *Shh* positive cells represented in single and merged channels with the nuclear marker DAPI (blue). Percentage of HuC/D^+^ neurons expressing different level of Shh mRNA density was quantified in the ARC, the VMH and the dorsomedial hypothalamic nucleus (DMH). Mean ± SEM. Density of Shh mRNA was estimated by RNAscope from *N* = 3 animals. More than 100 HuC/D-expressing cells were counted per animal in each of the three regions analyzed (**E**). *DG* dentate gyrus of the hippocampus, *3V* third ventricle, *ME* median eminence. Scale bars: **A** = 200 µm; **B**–**D** = 100 µm; **D1**–**D2** = 20 µm; all magnifications = 20 µm

**Fig. 3:**
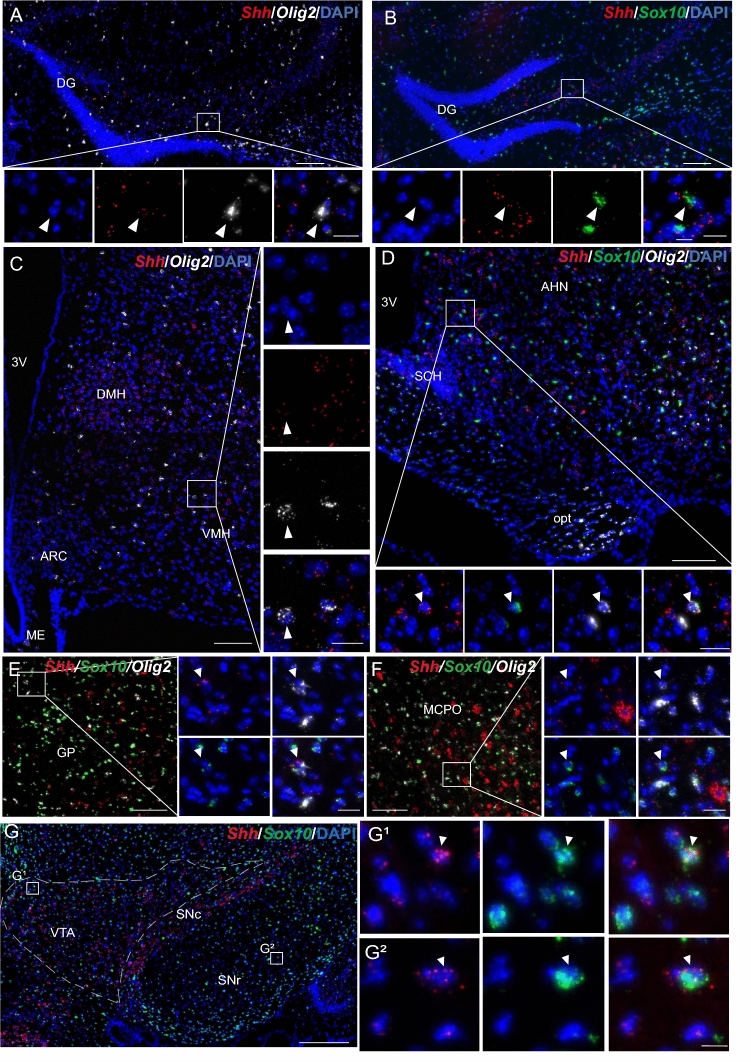
Representative images of multiplex fluorescent in situ hybridization of *Shh* (red) with the oligodendroglial markers *Sox10* (green) and *Olig2* (white) in the mouse hippocampus (**A**, **B**), hypothalamus (**C**, **D**), in the globus pallidus (GP) (**E**) and the magnocellular preoptic nucleus (MCPO) (**F**) of the pallidum, and in the midbrain (**G**). *Shh* is detected in *Sox10* or *Olig2* positive cells scattered in these brain regions as shown in the ventromedial hypothalamic nucleus (VMH) (**C**), the sub-paraventricular zone above the anterior suprachiasmatic nucleus (SCH) of the hypothalamus (**D**), and the ventral tegmental area (VTA) and the substantia nigra pars reticulata (SNr) (**G1**–**G2**). Arrowheads show *Shh* positive cells represented in single and merged channels with the nuclear marker DAPI (blue). White squares are magnified below the main panel (**A**, **B**, **D**) and on the side of the main panel (**C**, **E**–**G**). *DG* dentate gyrus of the hippocampus, *3V* third ventricle, *ME* median eminence, *ARC* arcuate hypothalamic nucleus, *DMH* dorsomedial hypothalamic nucleus, *opt* optic chiasma, *AHN* anterior hypothalamic nucleus, *SNc* substantia nigra pars compacta. Scale bars: **A**–**F** = 100 µm; **G** = 200 µm; **G1**–**G2** = 10 µm; all magnifications 20 µm

**Fig. 4:**
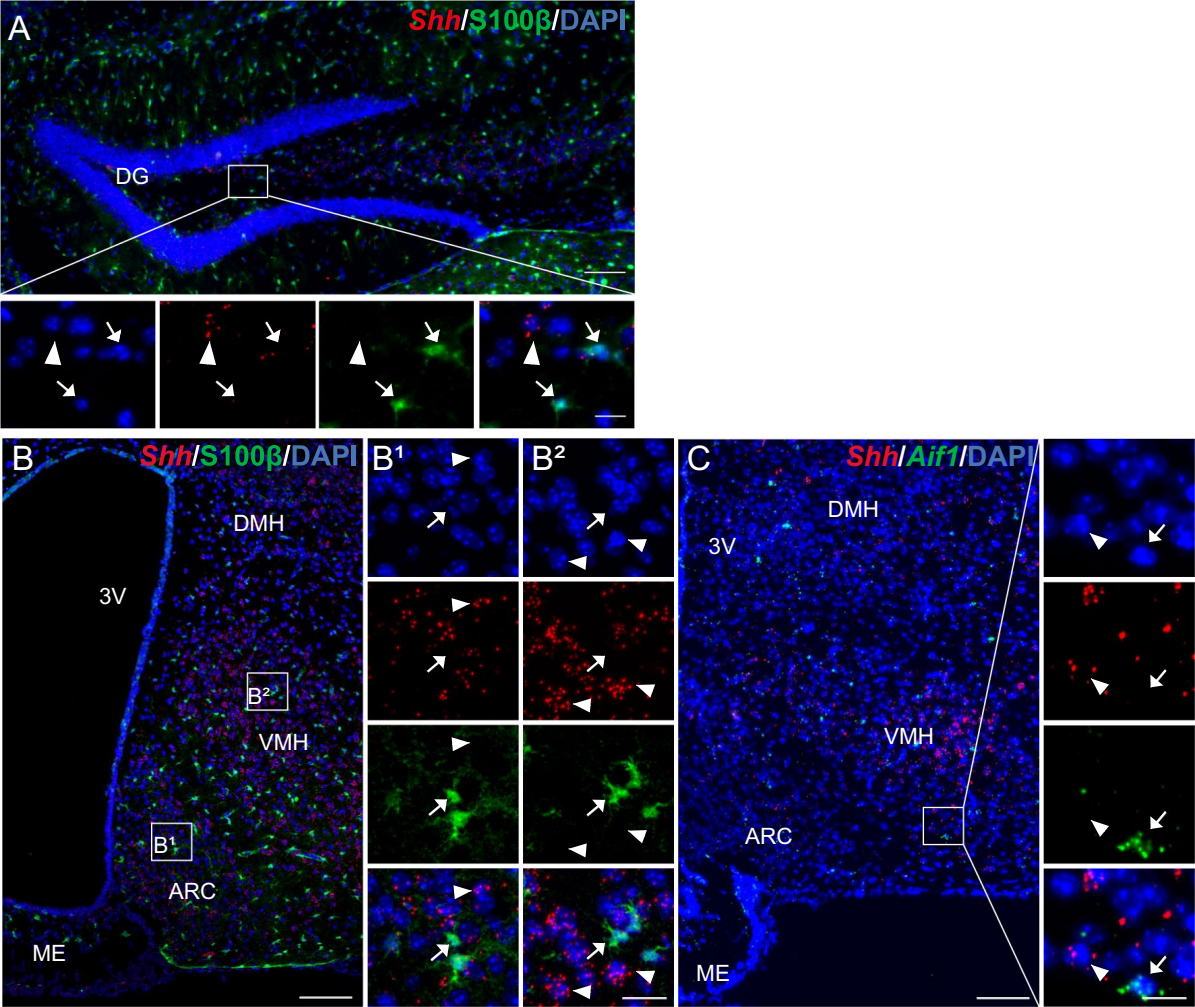
Representative images of multiplex fluorescent in situ hybridization of *Shh* (red) combined with immunofluorescence with the S100β astrocytic marker (green) (**A**, **B**) or with in situ hybridization with the *Aif1* microglia marker (green) (**C**) in the mouse hippocampus (**A**) and hypothalamus (**B**, **C**). *Shh* transcripts are not detected in S100β^+^ astrocytes as shown in the arcuate hypothalamic nucleus (ARC) (**B1**) and in the ventromedial hypothalamic nucleus (VMH) (**B2**), nor in *Aif1*^+^ microglia (**C**). Arrowheads show *Shh* positive cells and arrows show S100β^+^ or *Aif1*^+^ cells. B1 and B2 are magnifications of white boxes in **B**. White squares are magnified below the main panel (**A**) and on the side of the main panel (**C**) in merge and single channel with the nuclear marker DAPI (blue). *DG* dentate gyrus of the hippocampus, *3V* third ventricle, *DMH* dorsomedial hypothalamic nucleus. Scale bars: **A**–**C** = 100 µm; **B1**–**B2** = 20 µm; all magnifications = 20 µm

Further phenotyping of Shh mRNA-expressing cells in the mouse brain indicated that most of these cells were positive for the neuronal marker HuC/D, as shown in the cerebral cortex (Fig. 2A, B), the hippocampus (Fig. 2C) and the tuberal region of the hypothalamus (Fig. 2D), and recapitulated in Table 2. In the cortex and hypothalamus, activation of Shh signaling results in a different modulation of Gli1-3 in astrocytes (Tirou et al. 2021). Detailed analysis of Shh mRNA expression in HuC/D-positive cells revealed that in the hypothalamus, 34.0 ± 3.2% of neurons were positive for Shh transcripts in the ARC, 72.8 ± 4.3% in the VMH, and 42.5 ± 0.3% in the DMH (Fig. 2E, Table 3); whereas in the cerebral cortex, 29.6 ± 3.3% of the neurons (Table 3) expressed Shh mRNA. In the hypothalamus, Shh mRNA density was moderate to high in 4%–6% of ARC and DMH HuC/D-positive neurons and in 25% of VMH neurons (Fig. 2E). We also identified high Shh mRNA expression in GABAergic, cholinergic, nitrergic, and dopaminergic cells, as shown below and summarized in Tables 2 and 3. In the cerebral cortex, 95 ± 0.5% of the cells expressing Shh mRNA were HuC/D neurons (data not shown). Shh mRNA was also identified in a restricted cell population expressing the oligodendroglial markers *Sox10* or/and *Olig2* in all brain regions, as shown in the hippocampus (Fig. 3A, B) and in hypothalamic regions (Fig. 3C, D), in the globus pallidus and preoptic area (Fig. 3E, F), and in the dopaminergic region of the midbrain, including the ventral tegmental area (VTA), substantia nigra pars compacta (SNc) and reticulata (SNr) (Fig. 3G). Shh mRNA was undetectable in cells expressing the astroglial marker S100β as shown in the hippocampus (Fig. 4A) and in the tuberal region of the hypothalamus (Fig. 4B). Characterization of the microglial marker *Aif1* by smfISH on adult mouse brain sections revealed intense signals associated with scattered nuclei in brain regions, as shown in hypothalamic nuclei (Fig. 4C), in agreement with the distribution of IBA1-positive microglial cells (Lier et al. 2021). Shh mRNA was not identified in microglial cells expressing Aif1 transcripts, as shown in the hypothalamus (Fig. 4C).

**Table 2:** Distribution of *Shh* transcripts in the neuronal population of the mouse brain

Region	Nucleus	HuC/D	*Gad67*	*ChAT*	*nNOS*	TH
Cortex	Layer I	1^+^	1^+^	0^+^	1^+^	n.d
	Layer II/III	1^+^–3^+^	1^+^–3^+^	0^+^	1^+^–2^+^	n.d
	Layer IV	1^+^–2^+^	1^+^	0^+^	1^+^	n.d
	Layer V	1^+^–3^+^	1^+^–3^+^	0^+^	1^+^–3^+^	n.d
	Layer VIa	1^+^–3^+^	1^+^–2^+^	0^+^	1^+^–3^+^	n.d
	Layer VIb	1^+^–3^+^	1^+^–2^+^	0^+^	1^+^	n.d
Hippocampus	CA1	1^+^–3^+^	1^+^–3^+^	/	1^+^	n.d
	CA2	1^+^–3^+^	1^+^–3^+^	/	1^+^	n.d
	CA3	1^+^–3^+^	1^+^–3^+^	/	1^+^–3^+^	n.d
	Dentate gyrus					
	Granul cell layer	1^+^–3^+^	/	/	1^+^	n.d
	Polymorph layer	1^+^–2^+^	1^+^–2^+^	/	1^+^	n.d
	Molecular layer	1^+^–2^+^	1^+^–2^+^	/	1^+^	n.d
Striatum	Caudate putamen	1^+^–3^+^	1^+^–3^+^	0^+^	n.d	n.d
Pallidum	Globus pallidus	n.d	1^+^–4^+^	0^+^	n.d	n.d
	Magnocellular preoptic nucleus	n.d	1^+^–4^+^	1^+^–3^+^	n.d	n.d
Hypothalamus	Arcuate hypothalamic nucleus					
	Dorsal part	1^+^–3^+^	1^+^–3^+^	1^+^	1^+^–3^+^	n.d
	Ventral part	1^+^–3^+^	1^+^–3^+^	1^+^	1^+^–2^+^	n.d
	Ventromedial hypothalamic nucleus					
	Dorsomedial part	1^+^–3^+^	1^+^–3^+^	1^+^	1^+^–3^+^	n.d
	Central part	1^+^–3^+^	1^+^	1^+^	1^+^–3^+^	n.d
	Ventromedial part	1^+^–3^+^	1^+^	/	1^+^–3^+^	n.d
	Dorsomedial hypothalamic nucleus	1^+^–3^+^	1^+^–3^+^	1^+^	1^+^–3^+^	n.d
	Posterior hypothalamic nucleus	1^+^–3^+^	1^+^–3^+^	1^+^	1^+^	n.d
	Lateral hypothalamic nucleus					
	Anterior	n.d	1^+^–3^+^	/	n.d	n.d
	Posterior	1^+^–3^+^	1^+^–3^+^	0^+^	1^+^–3^+^	n.d
	Posterior	1^+^–3^+^	1^+^–3^+^	0^+^	1^+^–3^+^	n.d
	Median eminence	1^+^–2^+^	0^+^	/	0^+^	n.d
	Tuberal nucleus	1^+^–2^+^	1^+^–2^+^	0^+^	1^+^	n.d
	Anterior hypothalamic nucleus	n.d	1^+^–2^+^	/	n.d	n.d
	Sub-paraventricular zone	n.d	1^+^–3^+^	/	n.d	n.d
	Paraventricular hypothalamic nucleus	n.d	1^+^–2^+^	/	n.d	n.d
	Suprachiasmatic nucleus	n.d	1^+^	/	n.d	n.d
	Optic chiasma	n.d	/	/	n.d	n.d
Midbrain	Ventral tegmental area	n.d	n.d	n.d	n.d	1^+^–3^+^
	Substantia nigra, pars compacta	n.d	n.d	n.d	n.d	1^+^–3^+^
	Substantia nigra, pars reticulata	n.d	n.d	n.d	n.d	/
Pons	Mesencephalic trigeminal nucleus	n.d	1 +	4^+^	1^+^–3^+^	n.d
	Pontine central gray	n.d	1^+^–3^+^	1^+^–3^+^	1^+^–3^+^	n.d
	Motor trigeminal nucleus	n.d	1^+^–2^+^	4^+^	/	n.d
Medulla	Facial nucleus	n.d	1^+^–2^+^	4^+^	1^+^–3^+^	n.d
	Intermediate reticular nucleus	n.d	1^+^–2^+^	/	1^+^–3^+^	n.d
	Gigantocellular reticular nucleus	n.d	1^+^–3^+^	/	1^+^–3^+^	n.d
	Parapyramidal nucleus	n.d	1^+^	/	/	n.d
Cerebellum	Granule cell layer	n.d	1^+^	/	0^+^	n.d
	Purkinje cell layer	n.d	4^+^	/	/	n.d
	Molecular layer	n.d	1^+^	/	1^+^	n.d
	White matter	n.d	1^+^	/	/	n.d

Density of *Shh* mRNA was estimated by smfISH from N = 3–4 animals. 4 + : highest density, > 20 dots/cell; 3 + : high density, 10–20 dots/cell; 2 + : moderate density, 6–9 dots/cell; 1 + : low density, 2–5 dots/cell; 0 + : undetectable, 0–1 dot/cell; /: not relevant; n.d.: not determined

**Table 3:** Percentage of Shh transcripts expressing cells in HuC/D^+^, GABAergic *Gad67*^+^, and nitrergic *nNOS*^+^ neuronal populations of the mouse cortex and arcuate, ventromedial and dorsomedial hypothalamic nuclei

Region	*Shh* ^+^ cells (% ± SEM)^a^		
	HuC/D	*Gad67*	*nNOS*
Cortex	29.6 ± 3.3	5.9 ± 0.6	1.8 ± 0.2
Hypothalamus			
Arcuate	34.0 ± 3.2	36.1 ± 5.8	28.1 ± 3.7
Ventromedial hypothalamic nucleus	72.8 ± 4.3	n.d	56.4 ± 5.8
Dorsomedial hypothalamic nucleus	42.5 ± 0.3	56.2 ± 7.1	37.6 ± 1.1

The percentage is reported as mean ± SEM and was estimated by smfISH from *N* = 3 animals. More than 100 cells were counted per animal in each of the region analyzed. *n.d.* not determined

^a^Scattered *ChAT*^+^ cells expressing low *Shh* (1^+^) were observed in the arcuate, ventromedial hypothalamic nucleus, and the dorsomedial hypothalamic nucleus

### Shh mRNA is expressed in *ChAT* cholinergic neurons

**Fig. 5:**
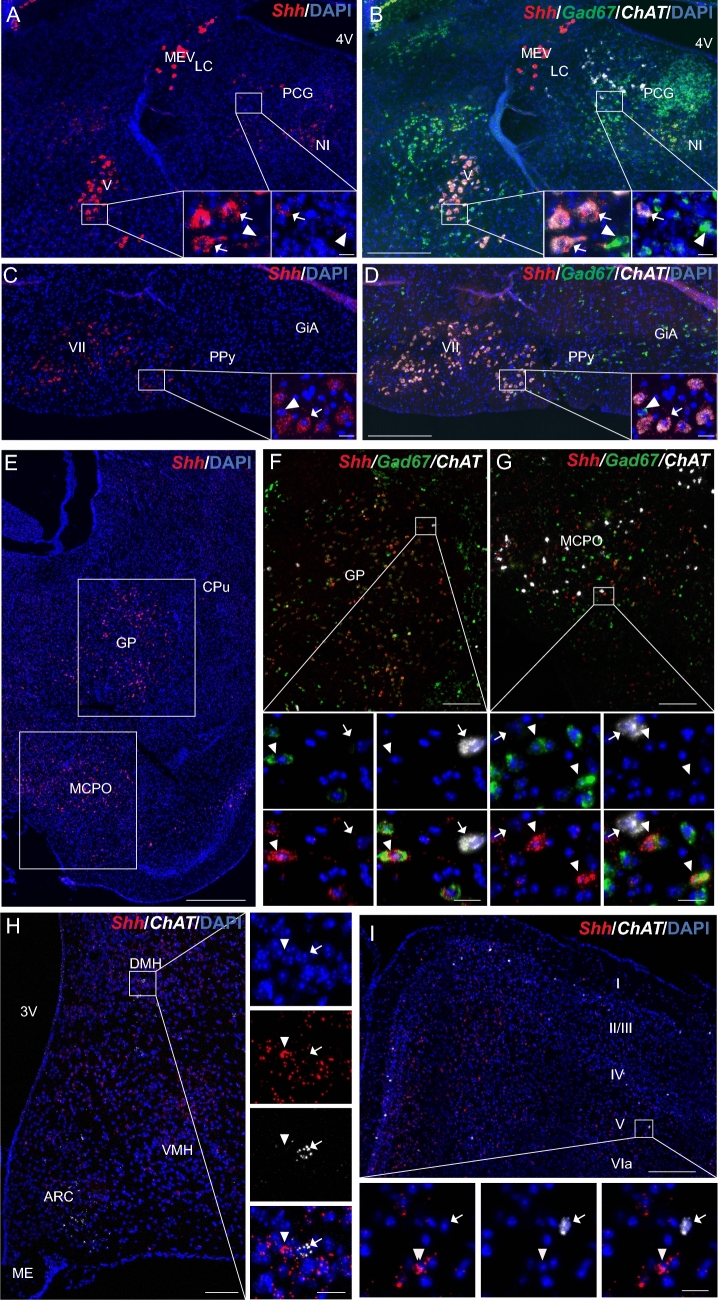
Representative images of multiplex fluorescent in situ hybridization of *Shh* (red) with the neuronal markers *Gad67* (green) and *ChAT* (white), together or alone, in the mouse hindbrain (**A**–**D**), the pallidum and the preoptic region (**E**–**G**), the tuberal region of the hypothalamus (**H**) and the cortex (**I**). *Shh* transcripts show wide expression with very high density level in *ChAT*^+^ cholinergic neurons (arrows) in the motor trigeminal nucleus (V), the pontine central gray (PCG) (**B**) and facial nucleus (VII) (**D**), while its intensity is lower in *Gad67*-expressing cells in those nuclei (arrowheads) (**B**, **D**). *Shh* transcripts show very high density level also in the globus pallidus (GP), the magnocellular preoptic nucleus (MCPO) and the dorsal part of the anterior amygdala area (AAD) (**E**). *Shh* is visualized at high levels in *Gad67*-expressing GABAergic neurons (arrowheads) but not cholinergic neurons expressing *ChAT* (arrows) in the GP, MCPO and AAD (**F**, **G**). *Shh* expression is very low in *ChAT*^+^ cholinergic neurons of the basal nuclei of the tuberal region of the hypothalamus, as shown in the dorsomedial hypothalamic nuclei (DMH) (**H**), while it is absent in *ChAT*^+^ cortical neurons (**I**). Arrowheads show *Shh*-expressing cells, while arrows show *ChAT*-expressing neurons (**H**, **I**). White squares are magnified on the corner of the main panel (**A**–**D**), below the main panel (**F**, **G**, **I**), or on the side (**H**), in merge and single channel with the nuclear marker DAPI (blue). *MEV* trigeminal nucleus, *4V* fourth ventricle, *LC* locus ceruleus, *NI* nucleus incertus, *PPy* parapyramidal nucleus, *GiA* anterior gigantocellular nucleus, *CPu* caudate putamen, *ARC* arcuate hypothalamic nucleus, *3V* third ventricle, *ME* median eminence. Scale bars: **A**–**D**, **I** = 200 µm; **E** = 500 µm; **F**–**H** = 100 µm; all magnifications = 20 µm

Shh mRNA expression in *ChAT*-expressing neurons in the adult mouse brain is shown in Table 2. *Shh* smfISH associated with the neuronal marker *ChAT* revealed a very high density of Shh transcripts in *ChAT-*positive cholinergic neurons located in the trigeminal mesencephalic and motor nuclei and in the facial nucleus (Fig. 5A–D), consistent with their expression in motor neurons, as previously reported (Traiffort et al. 1998, 1999, 2001). In addition, a moderate to high density distribution of Shh mRNA was observed in cholinergic neurons scattered in the pontine central gray (Fig. 5A, B), in the magnocellular preoptic nucleus of the pallidum **(**Fig. 5E, G; Table 2). Shh transcripts were also distributed at low density in *ChAT*-positive neurons located in hypothalamic nuclei, including the ARC, dorsomedial, and central parts of the VMH, DMH, and posterior hypothalamic nucleus (Fig. 5H; Table 2). Shh transcripts were undetectable in cholinergic neurons distributed in different layers of the cerebral cortex (Fig. 5I), in the caudate putamen and globus pallidus (Fig. 5F), or in the posterior part of the lateral hypothalamic nucleus or tuberal nucleus (Table 2).

### Shh mRNA is expressed in *Gad67* GABAergic neurons

**Fig. 6:**
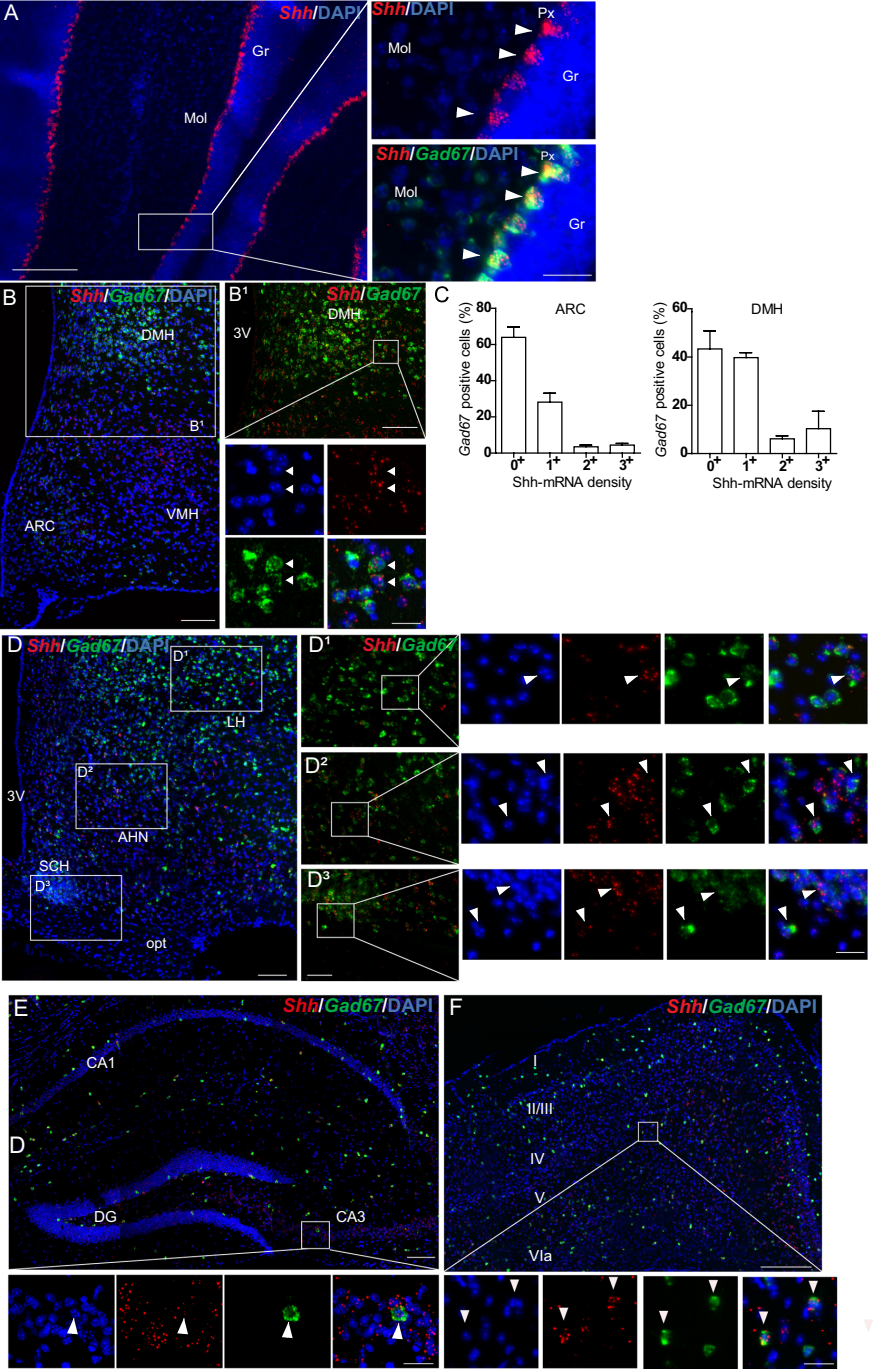
Representative images of multiplex fluorescent in situ hybridization of *Shh* (red) with the neuronal markers *Gad67* (green) in the mouse cerebellum (**A**), the tuberal region and the anterior region of the hypothalamus (**B**–**B**^**1**^, **D**–**D**^**3**^), the hippocampus (**E**) and the cerebral cortical layers (**F**). High level *of Shh* transcripts is detected in Purkinje cells (Px) expressing *Gad67* (**A**). In the hypothalamus (**B**), *Shh* transcripts show various density levels in *Gad67* GABAergic neurons as shown in the dorsomedial hypothalamic nucleus (DMH) (**B1**), and as quantified in the arcuate hypothalamic nucleus (ARC) and the DMH (**C**). Mean ± SEM. Density of Shh mRNA was estimated by RNAscope from *N* = 3 animals. *Shh* transcripts are also widely distributed in *Gad67*-expressing GABAergic neurons in the lateral hypothalamus (LH) (**D1**), the anterior hypothalamic nucleus (AHN) (**D2**), and in the suprachiasmatic nucleus (SCH), showing a less dense distribution at the level of the optic tract (opt) (**D3**). **D1**–**D3** are magnifications of boxed areas in **D**. In the hippocampus (**E**) and in the cortex (**F**), some *Shh*-expressing cells are scattered GABAergic neurons expressing *Gad67* as shown in the CA3 pyramidal cell layer (**E**) and in the cerebral cortical layer V (**F**). Arrowheads show *Shh* and *Gad67* double positive cells represented in merge and single channel with the nuclear marker DAPI (blue). White boxes are magnified on the side of the main panel for **A**–**D3**, and below the main panel for (**E**, **F**). *Mol* molecular layer, *Gr* granular layer, *3V* third ventricle, *VMH* ventromedial hypothalamic nucleus, *DG* dentate gyrus. Scale bars: **A** = 200 µm; **B**–**B1**, **D**–**D3**, **E**, **F** = 100 µm; magnifications = 50 µm (**A**) and 20 µm (**B1**, **D1**–**D3**, **E**, **F**)

The expression of Shh mRNA in *Gad67* GABAergic neurons in the adult mouse brain is reported in Table 2. Very high density of *Shh* transcripts in *Gad67*-expressing cells was observed in the pallidum (Fig. 5E–G) and in the Purkinje cell layer of the cerebellum in agreement with their expression in Purkinje cells (Fig. 6A). A high number of *Shh*^+^*Gad67*^+^ neurons were identified in the globus pallidus and the adjacent magnocellular preoptic nucleus (Fig. 5E–G**)**, whereas a more limited number was present in hypothalamic nuclei such as in the ARC, the VMH and the DMH (Fig. 6B) and in more anterior hypothalamic nuclei (Fig. 6D). Moderate to high density of Shh mRNA was also identified in scattered GABAergic neurons located in the CA1–CA3 pyramidal cell layers of the hippocampus (Fig. 6E), in the polymorph and molecular layer of the dentate gyrus, and in layers II/III, V, VIa and VIb of the cerebral cortex (Fig. 6F), in the caudate putamen (Fig. 5E–G)**,** in the pontine central gray **(**Fig. 5B), in the motor trigeminal and facial nuclei, and also in the intermediate and gigantocellular reticular nuclei (Fig. 5D). Low density of Shh mRNA was also detected in *Gad67*^+^ cells located throughout brain regions (Figs. 5A–D, 6B, D–F and Table 2). Quantification of Shh mRNA expression indicated that 36 ± 6% of GABAergic neurons were *Shh*^+^ in the ARC and 56 ± 7% in the DMH (Fig. 6C; Table 3); while in the cerebral cortex 6 ± 1% of *Gad*67^+^ neurons were expressing Shh transcripts (Table 3), with a total of 24 ± 3% of the cells expressing Shh mRNA being *Gad67*^+^ GABAergic neurons (data not shown). Shh mRNA was also evidenced in *Gad67*^+^ neurons in the VMH (Table 2) that occasionally populate this nucleus (Hrabovszky et al. 2012).

### Shh mRNA is expressed in *nNOS* nitrergic neurons

**Fig. 7:**
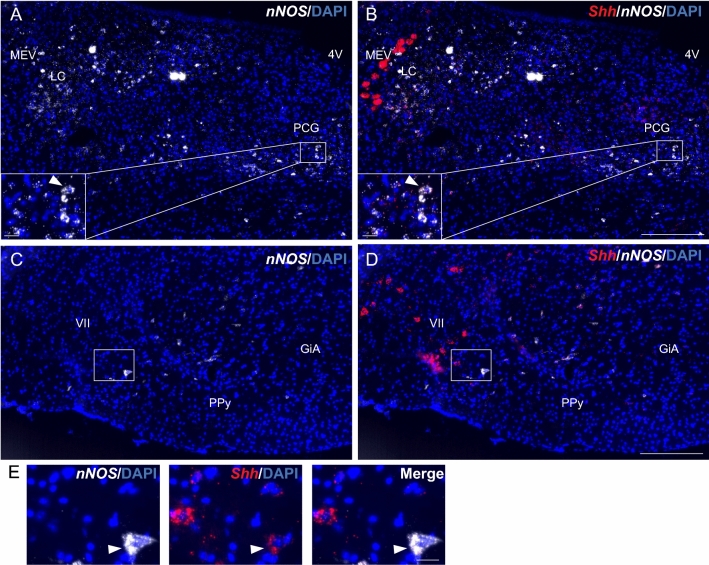
Representative images of multiplex fluorescent in situ hybridization of *Shh* (red) with the neuronal marker *nNOS* (white) in the mouse hindbrain. *Shh* transcripts are evidenced in cells expressing *nNOS* in the PCG (**A**, **B**) and in the facial nucleus (VII) (**C**–**E**) (arrowheads). **E** is a magnification of boxed areas in **C**, **D**. White squares are magnified on the corner of the main panels for **A**, **B**. Sections are counterstained using DAPI to visualize cell nuclei. *4V* fourth ventricle, *MEV* trigeminal nucleus, *LC* locus ceruleus, *PPy* parapyramidal nucleus, *GiA* anterior gigantocellular nucleus. Scale bars: **A**–**D** = 200 µm; all magnifications = 20 µm

**Fig. 8:**
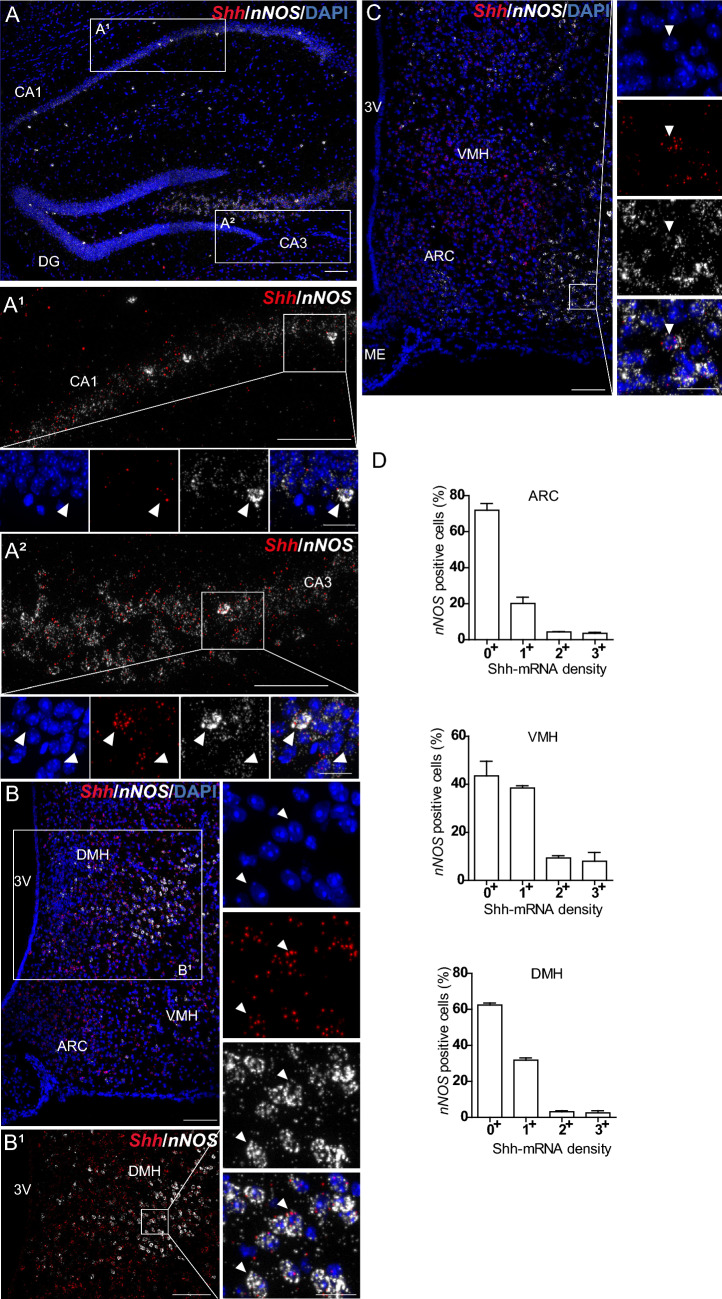
Representative images of multiplex fluorescent in situ hybridization of *Shh* (red) with the neuronal marker *nNOS* (white) in the mouse hippocampus and the tuberal region of the hypothalamus (**A**–**C**). *Shh* transcripts are detected at different intensity levels in nitrergic neurons expressing *nNOS* as shown in the CA1 (**A1**) and the CA3 (**A2**) pyramidal cells layers, respectively, with most of CA3 pyramidal cells expressing both *Shh* and *nNOS* transcripts (**A2**). *Shh* transcripts are widely distributed at different intensity levels in nitrergic neurons expressing *nNOS* located in the arcuate hypothalamic nucleus (ARC), the ventromedial hypothalamic nucleus (VMH) and the dorsomedial hypothalamic nucleus (DMH) **B**, **C**. Arrowheads show *Shh* and *nNOS* double positive cells. White square are magnified below the main panel for **A**, **B**, and on the side of the main panel for **B1**–**C**. Percentage of *nNOS*^+^ (**F**) neurons expressing different level of Shh mRNA density was quantified in the ARC, the VMH and the DMH. Mean ± SEM. Density of Shh mRNA was estimated by RNAscope from *N* = 3 animals. *DG* dentate gyrus, *3V* third ventricle, *ME* median eminence. Scale bars: **A**, **B**–**B1**, **C** = 100 µm; **A1**–**A2** = 50 µm; all magnifications = 20 µm

**Fig. 9:**
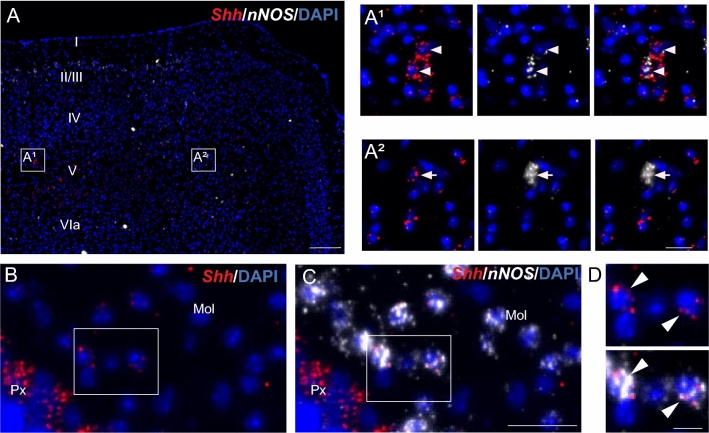
Representative images of multiplex fluorescent in situ hybridization of *Shh* (red) with the neuronal marker *nNOS* (white) in the mouse cerebral cortical layers (**A**) and the cerebellum (**B**–**D**). In all cerebral cortical layers, *Shh* intensity level is high in cells expressing a low intensity for *nNOS* transcripts (**A1**) (arrowheads), while it is low in cells expressing a high intensity level for *nNOS* transcripts (**A2**) (arrow). In the molecular layer of the cerebellum, *Shh* is distributed with a lower intensity level in nitrergic neurons expressing *nNOS* (**B**–**D**) (arrowheads). **D** is a magnification of boxed areas in **B** and **C**. White squares are magnified on the side of the main panels in merged channels with the nuclear marker DAPI (blue). *Mol* molecular layer, *Px* Purkinje cell layer. Scale bars: **A** = 200 µm; **B**, **C** = 30 µm; magnifications = 20 µm (**A**) and 10 µm (**B**, **C**)

Shh mRNA expression in *nNOS*-expressing neurons in the adult mouse brain is shown in Table 2. *Shh* smfISH combined with the *nNOS* marker identified consistent and robust expression of Shh mRNA in nitrergic neurons located in different brain areas. A moderate to high density of Shh transcripts was observed in cells located in the mesencephalic trigeminal nucleus and pontine central gray (Fig. 7A, B), in the intermediate reticular and gigantocellular nuclei, and in the facial nucleus in the medulla (Fig. 7C–E), in the CA3 pyramidal cell layer of the hippocampus, suggesting their expression in pyramidal neurons (Fig. 8A, A2), in various hypothalamic nuclei such as the ARC, VMH, DMH (Fig. 8B, C) and the posterior part of the lateral hypothalamic nucleus, and in cortical layers II/III, V, and VIa (Fig. 9A–A1). In the above regions, a low density of Shh mRNA was also detected in scattered cells expressing *nNOS*. We also identified a low density of Shh transcripts in nitrergic cells in hippocampal sparse cells located in the CA1 and CA2 pyramidal cell layers and in the dentate gyrus (Fig. 8A–A1), in the molecular layer of the cerebellum (Fig. 9B–D), in the tuberal and posterior hypothalamic nuclei of the hypothalamus (Fig. 8B–C), and in layers I, IV, and VIb of the cerebral cortex (Fig. 9A, A2). Quantification of Shh mRNA expression indicates that 28 ± 4% of nitrergic neurons are *Shh*^+^ in the ARC, 56 ± 6% in the VMH, and 38 ± 1% in the DMH (Fig. 8D; Table 3). In the cerebral cortex 1.8 ± 0.2% of *nNOS*^+^ neurons are *Shh*^+^ (Table 3) with a total of 6 ± 1.5% of the cells expressing Shh mRNA being *nNOS*^+^ nitrergic neurons.

### Shh mRNA is expressed in tyrosine hydroxylase-expressing dopaminergic neurons

**Fig. 10:**
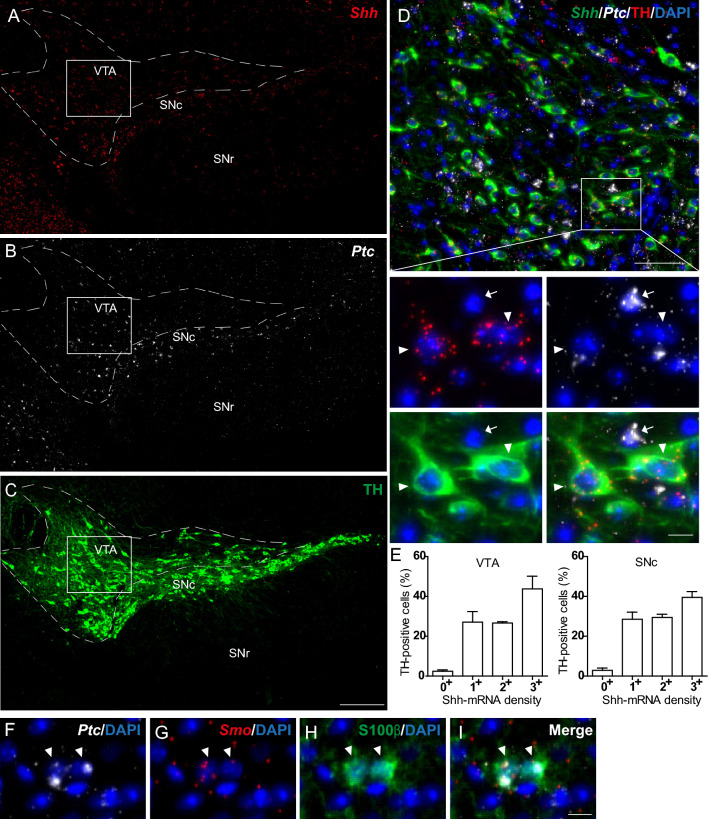
Representative images of fluorescent in situ hybridization of *Shh* (red) combined with immunofluorescence for the dopaminergic marker tyrosine hydroxylase (TH) (green), or with in situ hybridization of its receptor *Ptc* (white)*,* in the mouse midbrain. *Shh* and its receptor *Ptc* transcripts are widely distributed in the ventral tegmental area (VTA), delimited by the white dashed line, the substantia nigra pars compacta (SNc) and the substantia nigra pars reticulata (SNr) (**A**, **B**). Dopaminergic neurons, identified through TH-immunolabelling, are localized in the VTA and the SNc but not in the SNr (**C**). *Shh* transcripts are detected with different intensity levels in TH-expressing neurons (arrowheads), while *Ptc* transcripts are low in TH^+^ cells and higher in TH^−^ cells (arrows), as exemplified in the VTA (**D**). **D** is the magnification of the boxed area in **A**–**C**. Percentage of TH^+^ neurons expressing different level of Shh mRNA density was quantified in the VTA and SNc. Mean ± SEM. Density of Shh mRNA was estimated by RNAscope from *N* = 3 animals. More than 100 TH-expressing cells were counted per animal in each of the two regions analyzed (**E**). Astrocytes labeled by the astrocytic marker S100β (green) express both *Ptc* and the transducer of the Hh signaling pathway *Smo* (red) detected by fluorescent in situ hybridization, as indicated by arrowheads in a magnification of the SNc (**F**–**I**). Sections were counterstained with DAPI (blue) to visualize cell nuclei and identify the morphological limits of each structure. Scale bars: **A**–**C** = 200 µm; **D** = 50 µm; **F**–**I** and all magnifications = 10 µm

Shh mRNA expression in the hindbrain dopaminergic neurons is shown in Table 2. *Shh* smfISH combined with immunofluorescence of the neuronal tyrosine hydroxylase (TH) marker revealed a broad distribution of Shh mRNA in dopaminergic neurons in both the VTA and SNc (Fig. 10A, C), in agreement with a previous report based on a reporter mouse line (Gonzalez-Reyes et al. 2012; Turcato et al. 2022). Detailed analysis indicated that almost all TH-positive neurons expressed Shh transcripts at low to high density in the VTA (98 ± 6%) and in the SNc (97 ± 4%). Interestingly, a large proportion of TH-positive dopaminergic neurons expressed a high density of Shh mRNA (44 ± 6% in the VTA and 40 ± 3% in the SNc) (Fig. 10A, C–E). *Shh* smfISH combined with *Ptc* revealed co-expression of the Shh receptor with Shh in TH-positive neurons. However, the highest expression of Ptc was identified in scattered cells surrounding TH neurons in both the VTA and the SNc (Fig. 10A–D). SmfISH of *Ptc* and *Smo*, combined with immunofluorescence of the astroglial marker S100β, revealed that these cells were astrocytes and also expressed the Hh signaling pathway transducer Smo (Fig. 10F–I).

## Discussion

**Fig. 11:**
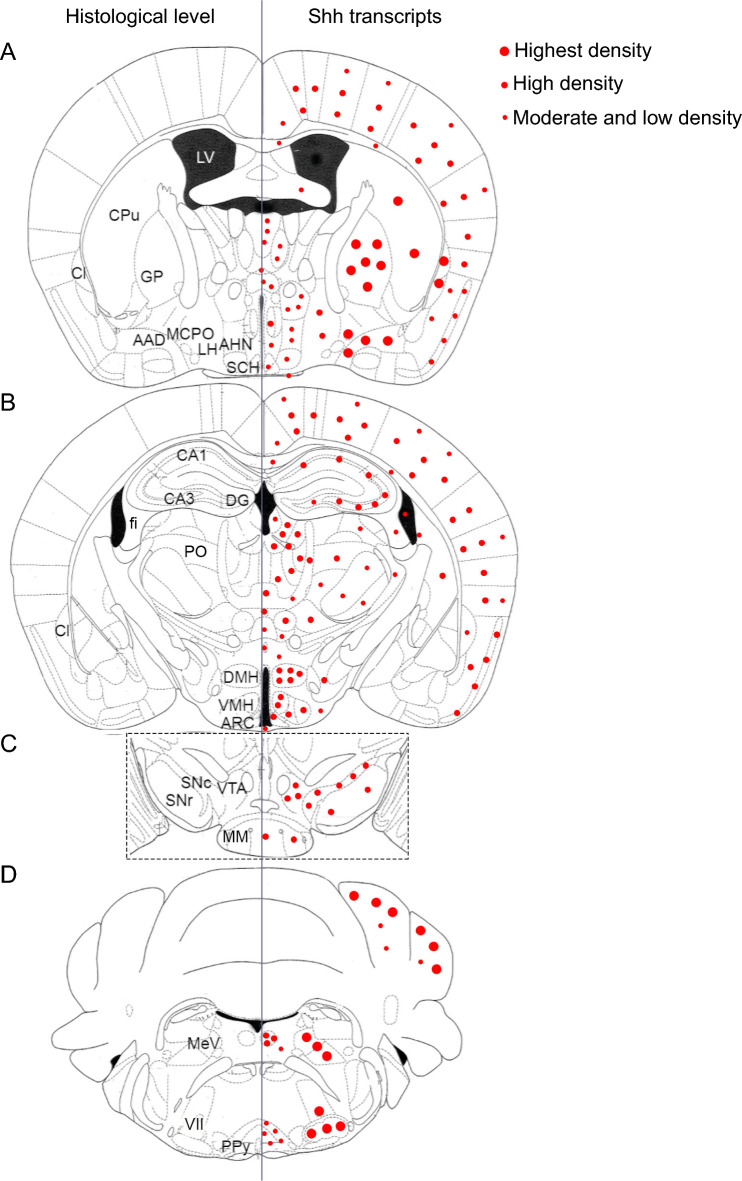
Line drawings of coronal sections of the adult mouse brain illustrating the distributions of Shh transcripts, indicated by red dots. These data are derived from ISH experiments conducted with Shh probe as described in Figs. 1, 2, 3, 4, 5, 6, 7, 8, 9, 10 and Table 1. The left column illustrates the histological level of neuroanatomical structures. VII, facial nucleus, *AAD* anterior amygdala area, dorsal, *AHN* anterior hypothalamic nucleus, *ARC* arcuate hypothalamic nucleus, *Cl* claustrum, *CPu* caudate putamen, *DG* dentate gyrus, *DMH* dorsomedial hypothalamic nucleus, *fi* fimbria, *GP* globus pallidus, *LH* lateral hypothalamic nucleus, *LV* lateral ventricle, *MCPO* magnocellular preoptic nucleus, *MeV* mesencephalic trigeminal nucleus, *MM* mammillary body, *PO* posterior thalamic nucleus, *PPy* parapyramidal nucleus, *SCH* suprachiasmatic nucleus, *SNc* substantia nigra pars compacta, *SNr* substantia nigra pars reticulata, *VMH* ventromedial hypothalamic nucleus, *VTA* ventral tegmental area. Bregma levels (mm): **A**, − 0.46; **B**, − 1.70; **C** − 3.08; **D**, − 5.68

Here, by further exploring the brain distribution of Shh, Dhh, and Ihh transcripts using smfISH (Tirou et al. 2020), we identified much broader expression of Shh mRNA than originally reported; whereas, Dhh and Ihh signals were not detectable (see summary Fig. 11A–D). Our current data unequivocally identify Shh transcripts in various populations of neurons as well as in a restricted population of oligodendroglial cells, both distributed in almost all brain regions. Astrocytes have been proposed as the major Shh-responsive cells in the adult rodent brain (Garcia et al. 2010, 2018; Ruat et al. 2015; Allahyari et al. 2019; Hill et al. 2019; Tirou et al. 2021; Wang et al. 2021). Thus, Shh synthesized and released by these neuronal and oligodendroglial cells is expected to regulate Hh signaling in astrocytes in these regions. We unequivocally identified Shh mRNA in GABAergic (*Gad67*^+^), cholinergic (*ChAT*^+^), nitrergic (*nNOS*^+^), and dopaminergic (TH^+^) neurons, and suggest its expression in other populations of neurons, including glutamatergic neurons in the cerebral cortex and in hypothalamic nuclei. These results are in agreement with previous reports describing Shh-transcript expression in GABAergic, cholinergic, dopaminergic, and some glutamatergic neurons using digoxigenin-labeled riboprobes (Traiffort et al. 1998; Traiffort et al. 2001; Charytoniuk et al. 2002; Lai et al. 2003; Machold et al. 2003; Loulier et al. 2006; Angot et al. 2008; Desouza et al. 2011; Ihrie et al. 2011; Ferent et al. 2013a; Eitan et al. 2016; Sanchez and Armstrong 2018; Sanchez et al. 2018; Gonzalez-Reyes et al. 2019; Rivell et al. 2019) or reporter lines (Garcia et al. 2010; Ihrie et al. 2011; Sanchez et al. 2018). Our present work identifies for the first time to our knowledge, *Shh* expression in nNOS neurons in several brain regions including the cerebral cortex, hippocampus and hypothalamus. Interestingly, neuronal *Shh* expression occurs at different levels of intensity, suggesting that Shh signals are tightly regulated in these cells. We also extend our previous observation of oligodendroglial Shh by reporting the presence of Shh mRNA in cells expressing the oligodendroglial markers *Sox10* or/and *Olig2* in all brain regions.

nNOS is responsible for the production of the radical gas nitric oxide (NO), which can act as an autocrine or paracrine messenger. Among the transduction pathways triggered by NO, activation of guanylate cyclase initiates transcription of various genes involved in multiple physiological processes, including modulation of synaptic transmission. A link between Shh signaling and NO has been established by a series of observations reporting that cGMP analogs increase the Shh differentiation response on chick neuronal explants (Robertson et al. 2001) while reduced NO levels in immature granule cell precursors (GCPs) of the outer granule cell layer during postnatal cerebellar development increase Ptc expression and impair GCP migration (Haag et al. 2012). The expression of Hedgehog-interacting protein (Hip), a negative regulator of Shh, in nNOS-positive neurons in the rodent brain (Loulier et al. 2005) also argues for a close regulation between NO and Shh signals. The presence of Shh transcripts in nNOS neurons suggests that, in these cells, Shh signals may be under the control of nNOS activity. Interestingly, the interaction of nNOS with Sox2 in the nucleus has been proposed to promote neuronal Shh transcription and to protect neurons from glutamate-induced excitotoxicity (Zhang et al. 2016). Alternatively, Shh may regulate nNOS activity through its action on local astrocytic Hh signaling and the release of glutamate and ATP factors, as demonstrated in cerebellar astrocytes (Okuda et al. 2016). Whether NO controls Shh transcription, synthesis, secretion, or degradation and whether Shh production attenuates or exacerbates the neurotoxicity associated with excessive NO production deserves further investigation.

In cerebral cortical layers, nNOS neurons have been divided into two types of inhibitory GABAergic neurons based on the intensity of nNOS immunoreactivity and NADPH diaphorase staining. Type 1 cells express high levels of nNOS immunoreactivity and NADPH diaphorase activity, are mainly found in layers V and VI (Oermann et al. 1999; Vercelli et al. 2000; Wiencken and Casagrande 2000; Barbosa et al. 2001; Lee and Jeon 2005; Perrenoud et al. 2012) and correspond mainly to projection neurons activated during sleep (Gerashchenko et al. 2008; Tricoire and Vitalis 2012). In our study, they may correspond to cells that express the highest level of nNOS mRNA and express little Shh mRNA (Fig. 9A, A2).

Type II cells correspond to a more heterogeneous population of neurons distributed throughout the mouse cortical layers, exhibiting low nNOS immunoreactivity and diaphorase activity (Lee and Jeon 2005; Perrenoud et al. 2012). They classically correspond to small- to medium-sized neurons with less characterized functions and also include neurogliaform interneurons mediating inhibitory GABAergic effects on pyramidal cells (Karagiannis et al. 2009; Olah et al. 2009). In our study, type II cells may correspond to cells expressing a lower level of nNOS mRNA (Fig. 9A, A1). Interestingly, the intensity of Shh mRNA is low to high in these cells.

In the hippocampus, the distribution of *Shh*^+^*nNOS*^+^ cells scattered throughout the hippocampal layers is in agreement with the distribution of nitrergic interneurons identified by NADPH diaphorase staining or immunohistochemistry (Tricoire and Vitalis 2012). Whether Shh mRNA is expressed in different types of nNOS interneurons, including VIP, calretinin, parvalbumin, or somatostatin interneurons, needs further investigation. The presence of nNOS mRNA identified in our study in the CA3 pyramidal field is in agreement with the network of nNOS-immunoreactive neurons in the rat (Chong et al. 2019) and in the mouse (Cork et al. 1998).

In the hypothalamus, we observed that the broad distribution of nNOS identified here by smfISH is very similar to that reported by immunohistochemistry (Chachlaki et al. 2017). Interestingly, our analyses of double-labeled structures highlight that Shh and nNOS mRNAs are abundantly co-expressed in hypothalamic nuclei, consistent with a potential role for NO signaling in modulating Shh activity. In DMH and VMH, it has been reported that 85% of nNOS-immunoreactive neurons are glutamatergic while less than 15% are GABAergic (Chachlaki et al. 2017). Thus, our data support that Shh is expressed in a large population of glutamatergic neurons as 38–56% of nNOS-positive neurons in these nuclei also express Shh mRNA. However, the Shh GABAergic neurons observed in the ARC, VMH, and DMH are also consistent with *Shh* expression in nNOS-positive cells, which are almost all GABAergic in the ARC (Chachlaki et al. 2017). *Shh* was not expressed in the few cholinergic neurons that populate these nuclei. Further studies, such as single-cell RNA sequencing, to access the molecular identity of hypothalamic Shh cell types are needed to identify which groups of GABAergic and glutamatergic neurons these cells belong to in these nuclei (Campbell et al. 2017) and in the LHA (Mickelsen et al. 2019; Rossi et al. 2019).

What are the physiological functions mediated by Shh ligand in hypothalamus? Recently, genetic activation of Shh signaling in mouse hypothalamic astrocytes has been shown to increase sensitivity to blood glucose levels and to display a major role in counteracting metabolic defects associated with aging and obesity (Tirou et al. 2021). The broad expression of Shh transcripts in multiple hypothalamic neuronal populations identified here, together with the distribution of both the precursor and the aminoterminal active fragments of Shh in hypothalamic tissues (Tirou et al. 2021), suggest that Shh signaling in astrocytes may be regulated locally to control glucose metabolism.

In addition to being expressed in the above-mentioned nuclei, *Shh* is also abundantly expressed in other hypothalamic nuclei implicated in the regulation of energy homeostasis such as the lateral hypothalamic nucleus or the paraventricular nucleus (Chachlaki et al. 2017). However, the presence of *Shh* in GABAergic neurons in the suprachiasmatic nucleus (SCN) supports a role for the pathway in regulating circadian rhythm as recently proposed (Tu et al. 2023). Shh from these neurons may modulate Hh signaling pathway mRNAs that are altered in the SCN of *Clock* mutant mice (Wang et al. 2018) or the activity of the superior cervical ganglion to regulate Ptc transcription in the pineal gland (Borjigin et al. 1999).

Activation of Shh signaling through the injection of active Shh protein in rodent or primate models of Parkinson’s disease has been shown to preserve TH immunoreactive neurons in the SN and dopaminergic axons in the striatum, improving motor function (Dass et al. 2002; Tsuboi and Shults 2002; Dass et al. 2005). The source of Shh that could signal for mediating these effects in the mesostriatal system has been unclear. We now provide further evidence that a source of Shh is possibly dopamine neurons of the SNc and of the VTA since almost all of these neurons expressed *Shh* in the adult mouse brain. Indeed, expression of Shh transcripts were not detected by us in our first studies in rat using specific digoxigenin-labeled riboprobes (Traiffort et al. 1998, 1999) or in mouse (unpublished observations), and was not reported by others using reporter mouse lines (Ihrie et al. 2011). Shh protein has not been reported, to our knowledge, in dopaminergic neurons by immunohistochemistry in adult rodent brain using Shh antibodies (Machold et al. 2003; Ihrie et al. 2011; Ferent et al. 2013b). Consequently, the absence of protein expression analysis in this region makes it difficult to verify consistency between transcript density and protein, an observation that may also apply to other brain regions where Shh protein expression analysis is absent. However, evidence for *Shh e*xpression by all TH neurons was observed using X-Gal staining of sections from 3-month-old mice heterozygous for a conditional, gene expression tracer allele of Shh (Shh-nLacZ^C/+^) (Gonzalez-Reyes et al. 2012). Thus, the apparent discrepancy among these reports may reflect different sensitivity of reporters, of the mouse line constructs and of antibodies used. Of interest, genetic ablation of Shh in TH-expressing neurons was associated with motor deficits that might have resulted from loss of dopaminergic, cholinergic and fast spiking GABAergic neurons (Gonzalez-Reyes et al. 2012). It was postulated that GDNF was implicated in the inhibition of Shh expression from dopaminergic neurons. Our present data also indicate the presence of *Ptc* on dopaminergic neurons that was not identified previously, suggesting a possible cell autonomous mode of Shh signaling on dopaminergic neurons. However, we show that astrocytes in the VTA and the SNc expressed high levels of *Ptc* and *Smo* transcripts suggesting that Shh signaling in astrocytes might participate in dopaminergic neurons regulation in the healthy brain and might be involved in the complex mechanisms underlying their loss during Parkinson’s disease (Kery et al. 2020).

## Conclusion

Although the precise distribution of Shh peptides needs to be further investigated in the adult rodent brain, the present results reveal that the Shh molecule is expressed more widely than originally thought. The widespread neuronal distribution of Shh mRNA in GABAergic, cholinergic, dopaminergic, nitrergic, and probably glutamatergic neurons, and its restricted expression in a population of oligodendroglial cells in almost all areas of the brain, suggest that Shh is an important physiological cue for astrocytes, which are the major Shh-responsive cells in the brain. Thus, Shh from neuronal sources may act locally or after transport to act on specific astrocyte populations to mediate anti-inflammatory actions or regulate energy metabolism (Garcia 2021; Tirou et al. 2021). However, as the transcription factor Gli1 is not expressed in neurons, Shh could act on neuronal Ptc (Traiffort et al. 1999) through non-canonical signaling to regulate neuronal functions and potentially the expression of Shh and Shh regulators (Loulier et al. 2005; Allahyari et al. 2019). Future physiological studies should be undertaken to examine how Shh mRNA and peptides are regulated from neuronal and oligodendroglial sources in healthy and pathological states. Such studies could not only provide a better understanding of the functions of Shh signaling in normal and pathological states, but also identify new regulatory mechanisms that could be targets for new drugs.

### Supplementary Information

Below is the link to the electronic supplementary material.

Supplementary file1 (DOCX 21 KB)

## Data Availability

Contact the corresponding author to access the primary data material.
