# Supplementary material for: Characterization of Sonic Hedgehog transcripts in the adult mouse brain: co-expression with neuronal and oligodendroglial markers
Source: Brain Struct Funct. 2024 Feb 8;229(3):705–27. doi: 10.1007/s00429-023-02756-2 (PMC10978748; doi:10.1007/s00429-023-02756-2)
Supplement: Supplementary file 1 — Supplementary file1 (DOCX 21 KB) [file 429_2023_2756_MOESM1_ESM.docx]

**Supplementary information**

**Characterization of *Sonic Hedgehog* transcripts in the adult mouse brain:**

**Co-expression with neuronal and oligodendroglial markers**

**Authors:** Mariagiovanna Russo^1^, Giuliana Pellegrino^1^, Hélène Faure^1^, Linda Tirou^1^, Ariane Sharif^2^ and Martial Ruat^1*^

**Running title:** Phenotyping brain Shh mRNA using smfISH

**Affiliations:**

^1^ CNRS, Paris-Saclay University, UMR-9197, Neuroscience Paris-Saclay Institute, F-91400, Saclay, France

^2^ Univ. Lille, Inserm, CHU Lille, Laboratory of Development and Plasticity of the Neuroendocrine Brain, Lille Neuroscience & Cognition, UMR-S 1172, FHU 1000 days for health, Lille, France

^*^**Correspondence**: Martial Ruat, CNRS, UMR-9197, Neuroscience Paris-Saclay Institute, F-91400, Saclay, France. Tel: 33 1 69 82 36 41; e-mail: martial.ruat@cnrs.fr

**ACKNOWLEDGMENTS**

M. Russo is recipient of a doctoral grant from the French Ministry of Research and the Foundation for Medical Research. This work was supported by grants from Foundation ARSEP (Aide a Recherche Sclérose en Plaque) to M. Ruat.

**Table 1: Control and target probes used for mouse *In situ* Hybridization.**

| Probe | ACD Cat. No. | Gene Accession ID | Target region | Z pairs | Dilution; Fluorophore | Use/ specificity |
| --- | --- | --- | --- | --- | --- | --- |
| dapB | 320871 | [EF191515](http://www.ncbi.nlm.nih.gov/nuccore/EF191515) | 414 - 862 | 10 | Adjusted to target probes used in parallel | Neg Ctrl, dapB from Bacillus subtilis |
| Mm-Polr2a | 320881 | [NM_009089.2](http://www.ncbi.nlm.nih.gov/nuccore/NM_009089.2) | 2802 - 3678 | 20 |  | Pos Ctrl, (low moderate) C1 |
| Mm-PP1B |  | [NM_011149.2](http://www.ncbi.nlm.nih.gov/nuccore/NM_011149.2) | 98 - 856 | 15 |  | Pos Ctrl, (moderate high) C2 |
| Mm-UBC |  | [NM_019639.4](http://www.ncbi.nlm.nih.gov/nuccore/NM_019639.4) | 34 - 860 | 20 |  | Pos Ctrl, (high) C3 |
| Mm-Aif1-C2 | 319141-C2 | [NM_019467.](http://www.ncbi.nlm.nih.gov/nuccore/NM_019639.4)2 | 31--866 | 18 | 1/800; Cy5 | Pan-Aif1 probe |
| Mm-ChAT-C2 | 408731-C2 | [NM_009891.2](http://www.ncbi.nlm.nih.gov/nuccore/NM_009891.2) | 1090 - 1952 | 20 | 1/2500; Cy5 | Pan-ChAT probe |
| Mm-Dhh | 415031 | [NM_007857.4](http://www.ncbi.nlm.nih.gov/nuccore/NM_007857.4) | 1235 - 2319 | 20 | 1/800; Cy3 | Pan-Dhh probe |
| Mm-Gad1-C3 | 400951-C3 | [NM_008077.4](http://www.ncbi.nlm.nih.gov/nuccore/NM_008077.4) | 62 - 3113 | 15 | 1/3000; Fluo | Pan-Gad67 probe |
| Mm-Ihh | 413091 | [NM_010544.2](http://www.ncbi.nlm.nih.gov/nuccore/NM_010544.2) | 990 - 2336 | 20 | 1/800; Cy3 | Pan-Ihh probe |
| Mm-Nos1-C2 | 447091-C2 | [NM_016967.2](http://www.ncbi.nlm.nih.gov/nuccore/NM_016967.2) | 865 - 2384 | 20 | 1/2000; Cy5 | Pan-nNOS probe |
| Mm-Olig2-C2 | 402811-C2 | [NM_008957.2](http://www.ncbi.nlm.nih.gov/nuccore/NM_008957.2) | 2260 - 3220 | 20 | 1/1500; Cy5 | Pan-Olig2 probe |
| Mm-Ptch1-C2 | 314361 | [NM_009170.3](http://www.ncbi.nlm.nih.gov/nuccore/NM_009170.3) | 307 - 1197 | 20 | 1/1500; Cy5 | Pan-Patched probe |
| Mm-Shh | 435931-C3 | [NM_011437.1](http://www.ncbi.nlm.nih.gov/nuccore/NM_011437.1) | 1087 - 2657 | 20 | 1/3000; Cy3 | Pan-Shh probe |
| Mm-Sox10-C3 | 437651 | [NM_008712.2](http://www.ncbi.nlm.nih.gov/nuccore/NM_008712.2) | 2 - 1097 | 20 | 1/1500; Fluo 1/3000; Cy5 with TH | Pan-Sox10 probe |
| Mm-Smo | 318411 | [NM_176996.4](http://www.ncbi.nlm.nih.gov/nuccore/NM_176996.4) | 1385 - 2266 | 20 | 1/1500; Cy3 | Pan-Smo probe |

ACD reference and NCBI accession number (Gene Accession ID) are listed for each probe. “Z pairs” corresponds to the size of the double "Z" oligo probes designed to hybridize the target region of the transcript. Neg Ctrl: Negative Control, Pos Ctrl: Positive Control. Control probes are internal controls of the technique. They are run in each RNAscope experiment to asses sample RNA quality, tissue optimal permeabilization and signal-to-noise ratio and they are not shown in the results. The level of expected copy number is indicated in parenthesis. All probes are Pan probes, designed to detect all mRNA isoform. Cy3: TSA plus Cyanine 3, Cy5: TSA plus Cyanine 5, Fluo: TSA plus fluoresceine.

**Table 2: Primary antibodies used for immunohistochemistry**

| Antigen | Host/ isotype | Immunogen | Dilution | Type, Clone | Source | Cat. No. | RRID |
| --- | --- | --- | --- | --- | --- | --- | --- |
| HuC/D | mouse/ IgG2b | (*) | 1/50 | Monoclonal, 16A11 | Thermo Fisher | A-21271 | AB_221448 |
| S100β | Rabbit | S100 isolated from cow brain. | Ready to use | Polyclonal | Agilent/ DAKO | IR50461-2 | AB_2811056 |
| (TH) Tyrosine hydroxylase | mouse/ IgG1 | TH purified from rat PC12 cells | 1/50 | Monoclonal, LNC1 | Millipore | MAB318 | AB_572268 |

* Antibody isolated from a patient with paraneoplastic encephalomyelitis.

**Table 3: Association of *In situ* probes and antibodies**

| Probe | Antibody | Mice | Sagittal plane, Postion to Bregma |
| --- | --- | --- | --- |
| Ihh | - | 3 | -1.7 |
| Dhh | - | 3 | -1.7 |
| Shh | - | 3 | From -1.46 to -2.3 |
| Shh | HuC/D | 3 | 0.38 |
|  |  | 5 | -1.7 |
| Shh-Gad67-ChAT | - | 3 | -0.22 |
|  |  | 3 | -0.46 |
|  |  | 4 | -1.7 |
|  |  | 4 | -5.68 |
| Shh-nNOS | - | 3 | -1.7 |
|  |  | 3 | -2.06 |
| Shh-nNOS-Sox10 | - | 4 | -5.68 |
| Shh-Sox10 | TH | 3 | -3.08 |
| Shh-Ptc | TH | 3 | -3.08 |
| Shh-Sox10-Olig2 | - | 3 | -0.34 |
|  |  | 3 | -0.46 |
|  |  | 3 | -1.7 |
| Shh-Aif1 |  | 3 | -1.7 |
| Smo-Ptc | S100β | 3 | -3.08 |
